# Mapping cancer patient online support groups: enhancing patient care in a low-middle income healthcare system

**DOI:** 10.1007/s00520-025-09535-1

**Published:** 2025-05-19

**Authors:** Fatma Bektash, Heba Hossam Ouda, Yasmine Hassan, Asmaa El-Sayed, Emad Shash

**Affiliations:** 1 Patient Education Unit, Breast Cancer Comprehensive Center, National Cancer Institute, Cairo University, Cairo, Egypt; 2 International Law and Political Science Department, American University in Cairo, Cairo, Egypt; 3 AstraZeneca, Washington, DC USA; 4 Quality Unit, Breast Cancer Comprehensive Center, National Cancer Institute, Cairo University, Cairo, Egypt; 5 Medical Oncology Department, National Cancer Institute, Cairo University, Cairo, Egypt

**Keywords:** Online support groups, Cancer patients, Facebook groups, Egypt

## Abstract

Cancer’s impact extends beyond the physical domain, affecting patients’ psychological, social, and financial well-being. Consequently, cancer support groups have a crucial role in guiding patients through their cancer journey and improving their quality of life. However, in Egypt, patient support groups remain undervalued and lack comprehensive visibility. This study aimed to address this gap by mapping patient online support groups in Egypt, categorizing them, and highlighting their importance in supporting patients and caregivers. The research includes examining online support groups and pages, particularly on Facebook. The results of our investigation indicate that 21% of the Facebook groups examined met the criteria for classification as qualified, another 21% were categorized as semi-qualified, while most of the groups, comprising 58%, were considered non-qualified. For the Facebook pages examined, organizational involvement through governmental, non-governmental, and charitable institutions qualified these pages as meeting the criteria for qualification. In contrast, the majority (70%) of the analyzed community pages were non-qualified, 30% were semi-qualified, and none met the criteria for qualification. This underscores the need for discernment when engaging with online resources. Furthermore, the study illuminates the role of various organizations, including hospitals, non-governmental organizations (NGOs), and pharmaceutical companies, in supporting cancer patients, especially through online support groups. Despite these efforts, comprehensive review articles on Egyptian patient support groups are lacking, making this study a significant contribution to understanding and enhancing patient support in the fight against cancer.

**Supplementary Information:**

The online version contains supplementary material available at 10.1007/s00520-025-09535-1.

## Background

### Cancer statistics

Cancer is a significant public health issue and a worldwide challenge, with the burden projected to increase to 28.4 million cases in 2040 (a 47% rise from 2020) [1–3]. Cancer ranks among the primary contributors to global mortality. World Health Organization (WHO) states that in 2020, cancer was responsible for almost 10 million fatalities worldwide, equating to approximately one out of every six deaths [4].

Egypt had an estimated 134,632 new cases of cancer and 89,042 cancer-related deaths in 2020, out of a total population of over 100 million people [5]. According to the Global Cancer Observatory (GLOBOCAN) report in 2020, the cancers with the highest 5-year prevalence rates in Egypt across all age groups are breast cancer (61,160 cases), liver cancer (28,977 cases), bladder cancer (26,986 cases), Non-Hodgkin lymphoma (19,096 cases), leukemia (14,274 cases), brain and central nervous system cancer (11,470 cases), and prostate cancer (10,523 cases) [5]. Along with this high cancer burden, there are pertinent healthcare system challenges in Egypt, including the increased incidence of cancer, the lack of unified national guidelines for the treatment of cancer, the increasing costs of cancer treatment, and the lack of awareness [6].

### Cancer’s multifaceted impact on patients

Cancer exerts a substantial influence on the quality of life experienced by patients, impacting various domains. These affected domains encompass the physical, psychological, social, and financial aspects, and their extent can be contingent upon the specific type and stage of cancer [7–9]. The ramifications of these effects extend beyond the perspective of patients and can also exert an influence on their caregivers [7].

In the physical health domain, effects include pain, fatigue, motor deficits, and sleeping difficulties. Cancer patients report independence-related problems as difficulties in daily activities, housework, and the ability to work. Cancer patients experience a decrease in social activities, family functioning, social support, and social well-being. Some also report reduced sexual functioning. Psychological health is also affected by cancer as patients suffer from anxiety, depression, and difficulty concentrating [10].

Individuals who have been diagnosed with cancer often experience significant unfulfilled needs, particularly when it comes to emotional assistance and access to medical knowledge. A lot of patients express the necessity of having medical information about treatment, future prognosis, and recovery prospects [7].

### Role of patient support groups

Every day, numerous individuals receive a cancer diagnosis, leading to significant demand for assistance and encouragement in navigating the challenges impacted by their diagnosis. Often, healthcare professionals (HCPs), social workers, and case managers are overwhelmed with their existing responsibilities [11, 12]. Indeed, patient support groups enable the exchange of personal journeys and mutual understanding, subsequently alleviating stress, and often provide a comfortable space to express feelings and experiences that might be challenging to share with family and friends. Online patient support groups can be categorized into two main types: peer-led or self-help groups and professional-led groups. Peer-led groups are managed by the members themselves, whereas professional-led groups are facilitated by qualified professionals such as counselors, social workers, or psychologists. These experts guide the discussions and interactions among participants, providing a structured environment for support and exchange [13].

The services rendered by patient support groups can yield advantages not only for patients, but also for caregivers, medical practitioners, and other HCPs. This efficacy stems from their pivotal role in furnishing essential support, encompassing aiding patients and caregivers in accessing pertinent educational resources, establishing credibility, and remaining abreast of pertinent advancements in medical innovation germane. Such facilitation is imperative due to the challenges faced by patients and their caregivers in effectively navigating the substantial volume of information and resources readily accessible to them [14, 15].

A psycho-educational program was conducted in the outpatient department of the clinical oncology center at the Nasser Institute Hospital in Egypt to show its impact on breast cancer patients. According to the findings, there was a notable decrease in anxiety and depression levels in patients following the program. Prior to the program, over half (52%) of the subjects experienced moderate anxiety and nearly one third (32%) experienced severe anxiety; also, about half (48%) reported severe depression, and over one third (34%) reported moderate depression. However, these figures decreased significantly to 15% and 8% for moderate and severe anxiety, respectively (*P* < 0.001), while severe and moderate depression decreased to 14% and 24%, respectively (*P* < 0.001) [16].

### The rise of online patient support groups

The increasing prominence of online support groups can be attributed to the rapid progression of technology. An online support group is defined as a digital platform accessible through diverse computer-mediated communication mediums, including laptops, smartphones, and desktop computers. Particularly noteworthy are social media platforms such as Facebook, which exemplify thriving hubs where these digital support groups flourish. These virtual communities allow individuals to participate in constructive dialogues and connect with others confronting similar challenges or concerns [17].

According to a survey done by the Egyptian Information and Decision Support Center (IDSC) in 2022, Facebook is the most used social media platform in Egypt. Among the 1002 Egyptian adults aged 18 and above that were surveyed, 95.9% of the respondents reported using Facebook, whether frequently, occasionally, or infrequently, and only 3.5% said that they don’t use Facebook [18].

The increased number of people receiving cancer diagnoses creates a need for support in facing associated challenges. In the context of Egypt and within the scope of our current awareness, support groups appear to be undervalued, leading to limited benefits for cancer patients. One contributing factor to this situation is the absence of comprehensive mapping or prominent visibility of these support groups in Egypt. Another factor is that many of these groups or pages lack supervision by HCPs, which raises questions about their reliability and trustworthiness. Consequently, the primary objective of our study is to develop a comprehensive map of patient support groups in Egypt, thereby facilitating better identification and utilization of their services for the benefit of patients. Given that Facebook is the most commonly used platform in Egypt, we opted to employ it in mapping patient support groups, along with their subsequent categorization. This approach was chosen to maximize the potential benefits derived from these groups.

## Methods

To map patient support groups and pages in Egypt, we conducted a comprehensive search and social listening on Facebook from April 2023 to September 2023. This timeframe was chosen to capture recent and relevant activities on social media, ensuring sufficient data for analysis and reflecting current trends in patient support initiatives. This approach aligns with methodologies used in similar studies examining how patients with rare conditions seek information and support through social media, as in a study by Marina L. Reppucci et al. (2022) [19]. We utilized a set of predefined terms in their translated Arabic form, including “cancer”, “cancer support Egypt”, “oncology support”, “nutrition for cancer patients”, and “cancer psychological support”.

We then identified several groups and activities related to cancer and other medical conditions on Facebook. Subsequently, we classified these groups into two classes: general and specific. General support groups encompass a broader spectrum of healthcare concerns extending beyond cancer, while specific support groups are exclusively dedicated to cancer-related issues. The objective is to elucidate distinct attributes and operational approaches between these two classes, shedding light on their roles in patient support and empowerment.

Following, we conducted a selection process by evaluating multiple parameters to verify that the group/page adequately fulfilled the study’s objectives and requirements, namely:

- Group/Page Description: To comprehend the emphasis, objective, and type of support provided.
- Groups’/Pages’ Moderators: To assess their responses to members and seek out precise regulations for accepting and prohibiting certain posts to keep a well-regulated and constructive online atmosphere.
- Membership Size/Follower Counts: To select groups with a minimum of 1000 members.
- Activity Status: To select groups with regular postings within the last 6 months.

Medical information disseminated on patient support groups or pages available on social media platforms such as Facebook can lead to misinformation, especially since non-specialists contribute heavily to these sources. Individuals intending to engage with online support groups should exercise caution, as the trustworthiness of these platforms can vary. In light of these considerations, the selected groups or pages were then categorized into three tiers: qualified, semi-qualified, and non-qualified. This categorization was based on specific criteria, including frequency of posting, admins’ background, control over questions raised and posting, and the involvement of HCPs.

**Fig. 1:**
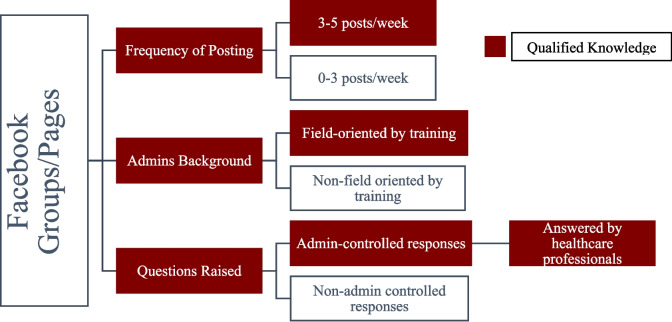
The criteria for qualified groups or pages

A group/page is deemed qualified if it meets the following standards: a minimum frequency of three to five posts per week, administrators with relevant backgrounds, and responses managed by the healthcare team, as shown in Fig. 1. Semi-qualified meet only two of these criteria, while non-qualified satisfy just one. The most critical criterion for qualification is the management of questions and responses; groups or pages are considered qualified when inquiries and posts are addressed by a healthcare team. In contrast, semi-qualified groups or pages may have responses from cancer survivors or volunteers, and non-qualified groups or pages lack input from a healthcare team entirely. Further parameters were also taken into account, such as the type of posts—whether general or specific— and the content of these posts. Types of posts include general knowledge related to the disease, disease misconception correction, early detection awareness, psychological support, responses to patients’ inquiries, free sessions/workshops, and donations.

## Results

**Fig. 2:**
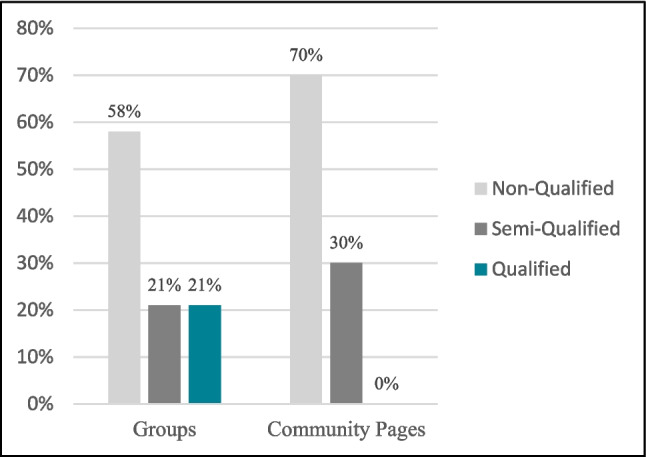
Qualification levels distribution. Distribution of qualification levels among Facebook entities indicates that only 21% of groups meet standards and 70% of community pages are non-qualified, highlighting limited access to trusted content for cancer patients

During our investigation on Facebook, we have identified several groups and pages dedicated to supporting cancer patients in Egypt. Our results show that the audience base of Facebook groups supporting cancer patients is larger than that of Facebook pages, as shown in Table 1. Among these entities, 21% of the Facebook groups were deemed qualified, 21% were classified as semi-qualified, while the majority, 58%, of the groups were considered non-qualified. This means that a significant portion of the Facebook groups did not meet the qualification criteria, while only a minority were semi-qualified or fully qualified. Consequently, the number of members in qualified groups is 15,700, which represents a low percentage—5.7%—of cancer patients receiving trusted content relative to the total number of members in the study groups. Regarding Facebook community pages, we found that the majority—70%—did not meet the qualification criteria, while 30% were semi-qualified, and none were considered qualified as elaborated in Fig. 2. In contrast, all governmental, non-governmental, and charitable organizations we investigated possessed the capability to disseminate valuable information.

**Table 1:** Members/followers distribution

	No. of members/followers	Members/followers percentages
Groups	274,199	59.09%
Community pages	189,800	40.91%

**Fig. 3:**
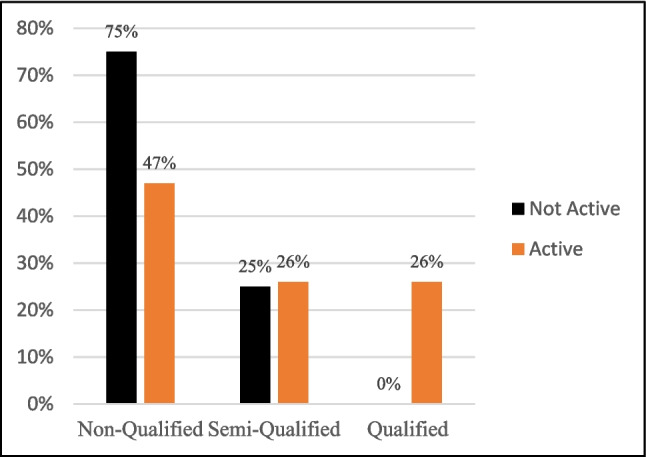
The activity of patient support entities on Facebook according to their qualifications. Activity analysis of patient support entities on Facebook shows that qualified groups account for 26% of overall active participation, while 75% of inactive entities are non-qualified

**Fig. 4:**
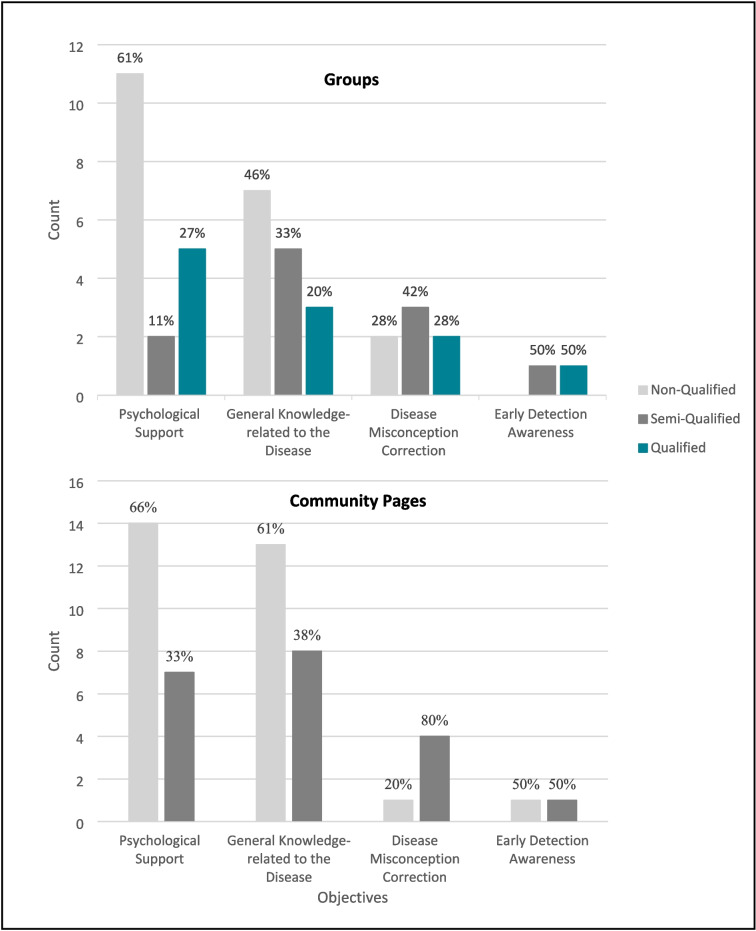
The main objectives of groups and community pages. Posting counts for main objectives across qualifications reveal that qualified groups mostly post about psychological support; however, 61% of this content originates from non-qualified groups. Awareness posts on early detection are equally shared by qualified and semi-qualified entities

Our investigations reveal positive insights. Firstly, all qualified groups were active as illustrated in Fig. 3. Secondly, all groups and community pages cover various objectives, indeed. The content published by the qualified groups includes psychological support, general knowledge about the disease, disease misconception correction, raising awareness about early detection, and responding to patients’ inquiries. Similarly, semi-qualified entities publish the same types of content and offer additional free sessions and workshops. Besides posting similar content to qualified and semi-qualified groups, non-qualified groups post about donations needed to help low-income patients. Although discussing different topics may benefit cancer patients, it is noteworthy that most posts providing psychological support and general knowledge come from non-qualified entities, as shown in Fig. 4. This highlights the need for more active participation from qualified entities to ensure the accuracy and reliability of disseminated information.

The administrators of qualified groups include oncologists, psychologists, oncology pharmacists, radiotherapy lecturers, and volunteers. In addition to these titles, semi-qualified groups include administrators who are cancer survivors, skincare specialists, physicians, scientists, and nutritionists. In contrast, non-qualified entities are managed mainly by volunteers, cancer survivors, and life coaches. Further findings of the studied groups and pages are presented in Supplementary Material 1 and Supplementary Material 2 documents, respectively. Each table furnishes information such as the starting date, the number of members or followers, administrators, content objectives, post types, activity status, and the overall qualification of the groups or pages.

## Discussion

Cancer represents a global public health challenge, with the number of diagnosed cases projected to rise to 28.4 million by 2040. In Egypt, this challenge is accompanied by healthcare system limitations, including widespread misinformation and insufficient public awareness about cancer. These factors add to the disease’s profound impact on patients’ quality of life, affecting their physical, psychological, social, and financial well-being. Support groups play a crucial role in addressing some challenges by reducing social isolation and meeting emotional needs. They provide patients with shared experiences, emotional support, and access to educational resources. Among these, online support groups have gained prominence due to their accessibility and cost-free nature.

Our findings highlight the critical need for cancer patients and caregivers to identify qualified sources of information when engaging with Facebook groups and community pages. Given the complexity of cancer-related content and the public’s limited ability to evaluate medical accuracy, we propose standardized criteria to distinguish reliable support groups from unqualified ones. To raise awareness, coordinated efforts should be made to conduct educational campaigns organized by support groups in collaboration with key stakeholders, including healthcare organizations, pharmaceutical companies, and regulatory bodies. These initiatives should focus on providing clear, unified guidelines for online support groups, in addition to providing proper training to the facilitators of these support groups and patient advocates to ensure they have the necessary skills and knowledge to offer emotional support and accurate scientific information. Furthermore, establishing a communication channel between online support groups and designated personnel, such as healthcare professionals and representatives from patient support programs, would likely improve the quality of information shared on these groups.

### Organizational involvement

The significance of organizational involvement, encompassing hospitals, non-governmental organizations (NGOs), and online support entities, within the patient support domain in Egypt, warrants considerable attention. Nevertheless, while support groups are available in many NGOs, there is a substantial requirement for cancer support groups in other sectors. Our research revealed a dearth of support groups on social media platforms and a limited presence of active support groups in governmental organizations. However, the Breast Cancer Comprehensive Center (BCCC) of the National Cancer Institute (NCI)—Cairo has initiated patient support efforts through the establishment of patient education and navigation units [20]. The BCCC comprises medical professionals, breast cancer survivors, and community leaders. This coalition collaboratively drives impactful changes in the lives of Egyptian women affected by breast cancer, while ensuring the dissemination of reliable information, meeting the standards set by our proposed qualification criteria. Similarly, Baheya’s Psychological Support Department focuses on the preservation of both the physical and mental health of patients. It achieves this by offering reliable information through various initiatives, including workshops and educational sessions.

The need for more of these supervised initiatives has been emphasized through a qualitative study conducted in October 2022 on 487 individuals, including both patients and their relatives, at the BCCC. The results revealed that 59.80% of individuals held erroneous beliefs regarding breast cancer. Moreover, only 3.00% of these individuals acquired their knowledge from the healthcare team, while the majority depended on potentially untrustworthy sources such as social media platforms, the Internet, caregivers, fellow patients, television, and radio. These findings point to an overreliance on non-verified sources for health information. Strategic large-scale dissemination of accurate information could contribute significantly to enhancing breast cancer awareness and helping individuals make informed health decisions [21]. Achiving this requires collaboration with key healthcare stakeholders, including pharmaceutical companies.

### Role of pharmaceutical companies in supporting cancer patient groups

Pharmaceutical companies not only provide essential cancer medications, but they also engage in patient care initiatives. Additionally, pharmaceutical companies share common goals with patient support groups; as a result, many companies establish partnerships and, in some cases, provide financial support to patient support groups. Both sectors collaborate to develop balanced, non-promotional information about medicines, offering clear explanations about their products, proper side-effect management, and adherence enhancement tools. Ongoing cooperation between health professionals, pharmaceutical companies, and cancer patient groups in the aforementioned areas is important for cancer patients, especially in clinical trials, medicines information, and treatment adherence [22].

For example, AstraZeneca actively collaborates with patient groups in oncology to establish early detection programs, and partners with governmental and non-governmental entities [23]. Moreover, the company showcases its commitment to supporting healthcare initiatives in Egypt through patient support programs and solutions, aligning with precision medicine principles and ensuring the prompt availability of innovative medicines to Egyptian patients [24].

## Conclusion

In conclusion, cancer’s multifaceted impact on patients spans physical, psychological, social, and financial dimensions, varying based on cancer type and stage. These effects also extend to caregivers. Physical symptoms and psychological challenges contribute to a complex cancer journey. Patient support groups serve as intermediaries, helping patients navigate complex information and offering emotional support. Initiatives like a psycho-educational program at the Nasser Institute Hospital showcased the impact of advocacy, and also, the significance of directing attention toward enhancing awareness and providing patients with accurate information from reputable sources, such as the research conducted at the BCCC-NCI. However, comprehensive review articles on Egyptian patient support groups are lacking. This study aimed to fill this gap by categorizing these groups and highlighting collaborations with healthcare institutions and pharmaceutical companies, such as AstraZeneca Egypt.

Given the identified gaps in the cancer patient support landscape in Egypt, a centralized database is needed to track participation rates in support groups and programs, as well as patient outcomes and feedback from group members. This will provide evidence of the long-term benefit of support groups. Moreover, our study only focused on Facebook support groups, and accordingly, a more comprehensive analysis of support groups, encompassing social media platforms other than Facebook as well as offline services.

The fight against cancer necessitates a multi-pronged approach involving patient advocacy, healthcare institutions, and industry partnerships. These collaborations reshape healthcare to better serve patients and families, providing essential information and resources. Patient support groups remain a vital aspect of compassionate care, empowering patients to confront cancer with resilience and optimism.

Supplementary information.

## Supplementary Information

Below is the link to the electronic supplementary material.

Supplementary file1 (DOCX 108 KB)

Supplementary file2 (DOCX 161 KB)

## Data Availability

The data that support the findings of this study are available from the corresponding author.
