# Supplementary material for: Mapping cancer patient online support groups: enhancing patient care in a low-middle income healthcare system
Source: Support Care Cancer. 2025 May 19;33(6):480. doi: 10.1007/s00520-025-09535-1 (PMC12089213; doi:10.1007/s00520-025-09535-1)
Supplement: Supplementary file 1 — Supplementary file1 (DOCX 108 KB) [file 520_2025_9535_MOESM1_ESM.docx]

**Article title:** Mapping Cancer Patient Online Support Groups: Enhancing Patient Care in a Low-Middle Income Healthcare System.

**Journal name:** Supportive Care in Cancer

**Author names:**

Fatma Bektash, Heba Hossam Ouda, Yasmine Hassan, Asmaa El-Sayed, Emad Shash^*^

***Corresponding author:**

- **Affiliation:** National Cancer Institute - Breast Cancer Comprehensive Center, Medical Oncology Department, Cairo University, Cairo, Egypt
- **E-mail address:** [emad.shash@nci.cu.edu.eg](mailto:emad.shash@nci.cu.edu.eg)

**Supplementary Material 1. Patient Support Entities on Social Media (Facebook) - Groups**

| Name | Starting date | No. of members | Admins | Objectives | Type of posts | Active/ Not active | Qualification |
| --- | --- | --- | --- | --- | --- | --- | --- |
| السرطان بدايه حياه 💪👈(معا أقوي(  Cancer is the beginning of a new life (together we are stronger)  <https://www.facebook.com/groups/463681420319556> / | August 17, 2012 | 49.8K | - Oncologists.  - Nutritionist.  - Patient survivors  - Volunteers. |  | Specific | Active | Semi-qualified |
| آلام السرطان والعلاج  Cancer pain and treatment  <https://www.facebook.com/groups/840026986088828> | June 26, 2015 | 9.4K | - Volunteers aim to support patients. |  | General | Active | Not qualified |
| مرضى السرطان  Cancer Patients  <https://www.facebook.com/groups/1764040937177660> / | August 26, 2016 | 6.8K | - Volunteer. |  | General | Active | Not qualified |
| محاربو السرطان  Cancer Fighters  <https://www.facebook.com/groups/1458472864215368> | March 31, 2017 | 31.5K | - Volunteers from different fields.  - Cancer survivors. |  | Both general and specific | Active | Not qualified |
| قصتي مع السرطان والأمل  My Story with Cancer and Hope  <https://www.facebook.com/groups/159859187890024> | June 26, 2017 | 32.3K | - Volunteers from different fields and countries.  - Patient survivors. |  | Both general and specific | Active | Not qualified |
| تجربتي مع السرطان  My Experience with Cancer  <https://www.facebook.com/groups/533902440283929> | September 12, 2017 | 9.2K | - Two surviving patients whose goal is to share their experience with others. |  | Specific | Not Active | Not qualified |
| أنا أقوى من السرطان  I am stronger than cancer  <https://www.facebook.com/groups/1455760634518209> | October 8, 2017 | 30.5K | - Oncologists.  - Pharmacists.  - Clinical nutritionists  - Patient survivors. |  | General | Active | Semi-qualified |
| علاج السرطان مصر  Cancer treatment in Egypt  <https://www.facebook.com/groups/AskDoctorOnline> | October 21, 2017 | 3.5K | - Two Oncologists and the Al-Ahram Center for Oncology Treatment. |  | Both general and specific | Active | Qualified |
| جروب مدد لدعم مرضى السرطان  Madad: Support group for cancer patients  <https://www.facebook.com/groups/330740737332977/> | January 31, 2018 | 1.3K | - Two psychologists.  - Two volunteers aim to support patients. |  | Specific | Active | Qualified |
| تحدي مرضي السرطان (استشارات)  Cancer Patients Challenge (Consultations)  <https://www.facebook.com/groups/177461012883556> / | March 15, 2018 | 13.9K | - Volunteers. |  | General | Active | Not qualified |
| السرطان بدايه مش نهايه جروب خاص لمحاربين وداعمين مرضى السرطان  Cancer is just the beginning, not the end - a special group for cancer warriors and supporters  <https://www.facebook.com/groups/324883998095456> / | October 15, 2018 | 9.5K | - Oncologists.  - Skincare specialist.  - Volunteers. |  | Specific | Active | Semi-qualified |
| كل ما يهمك عن أورام الثدي  Everything you need to know about breast tumors  <https://www.facebook.com/groups/171387757141851> | October 30, 2018 | 4.9K | - Oncology pharmacists.  - Oncologists. |  | General | Active | Qualified |
| كلنا ضد السرطان  We are all against cancer  <https://www.facebook.com/groups/361772418030761> | June 12, 2019 | 3.0K | - Two volunteers.  - Hairdresser who supports cancer patients by providing them with free services. |  | Both general and specific | Not Active | Not qualified |
| مرضى السرطان  Cancer Patients  <https://www.facebook.com/groups/457470988445074> | September 26, 2019 | 7.7K | - Civil engineer.  - Life coach. |  | Both general and specific | Active | Not qualified |
| قصص وتجارب مريضات السرطان  Stories and experiences of cancer patients  <https://www.facebook.com/groups/226620421704636> | February 9, 2020 | 28.4K | - Two volunteers. |  | Specific | Not Active | Not qualified |
| Fight Cancer  <https://www.facebook.com/groups/2782100455203979> | February 22, 2020 | 2.6K | - A consultant oncologist.  - Volunteer to manage the group. |  | General | Active | Semi-Qualified |
| Cancer Super Heroes  <https://www.facebook.com/groups/626275814604687> | March 20, 2020 | 3K | - Oncologists  - 2 Oncology pharmacists.  - Volunteers. |  | Both general and specific | Active | Qualified |
| كيف تتعايش مع السرطان  How to cope with cancer  <https://www.facebook.com/groups/541538353467650> | May, 2020 | 6.9K | - Five volunteers from different fields. |  | General | Active | Not qualified |
| هيا اصحبني نحو الشفاء من السرطان  Let's journey towards healing from cancer together  <https://www.facebook.com/groups/495576988070205> | December, 2020 | 3K | - Oncologist.  - Lecturer (radiotherapy). |  | General | Active | Qualified |
| الجمعية المصرية لأبحاث السرطان - Egyptian Association for Cancer research  <https://www.facebook.com/groups/139514011582838> / | June 21, 2021 | 1.2K | - Oncologists.  - Pharmacists.  - physicians/ scientists.  - Volunteers. |  | General | Not active | Semi qualified |
| نصائح وتثقيف لمرضي السرطان  Tips and education for cancer patients  [https://www.facebook.com/groups/870655383656497 /](https://www.facebook.com/groups/870655383656497%20/) | September 10, 2021 | 1.3K | - Volunteer. |  | General | Active | Not qualified |
| نظام غذائي لمرضى سرطان الثدي  Dietary plan for breast cancer patients  <https://www.facebook.com/groups/966847034184428> | December 4, 2021 | 5.2K | - Two volunteers from different fields. |  | General | Not Active | Not qualified |
| نصائح مهمه لمرضى السرطان و قبل إستئصال الثدى  Important tips for cancer patients and before breast removal surgery  <https://www.facebook.com/groups/523170329219587> / | April 14, 2022 | 1.9K | - Two volunteers. |  | Specific | Not Active | Not qualified |
| جمعية كانسر فايترز لدعم مرضي السرطان  Cancer Fighters Association for supporting cancer patients  <https://www.facebook.com/groups/526492605816778/> | May 7, 2022 | 7.4K | - patient survivors.  - volunteers. |  | Both general and specific | Active | Not qualified |

**Note:** The figures presented in this table were revised and current as of September 29, 2023.

General knowledge related to the disease.

Disease misconception correction.

Early detection awareness.

Psychological support.

Respond to patients' inquiries.

Free sessions/ workshops.

Help low-income patients financially (Donations).
