# Supplementary material for: Mapping cancer patient online support groups: enhancing patient care in a low-middle income healthcare system
Source: Support Care Cancer. 2025 May 19;33(6):480. doi: 10.1007/s00520-025-09535-1 (PMC12089213; doi:10.1007/s00520-025-09535-1)
Supplement: Supplementary file 2 — Supplementary file2 (DOCX 161 KB) [file 520_2025_9535_MOESM2_ESM.docx]

**Article title:** Mapping Cancer Patient Online Support Groups: Enhancing Patient Care in a Low-Middle Income Healthcare System.

**Journal name:** Supportive Care in Cancer

**Author names:**

Fatma Bektash, Heba Hossam Ouda, Yasmine Hassan, Asmaa El-Sayed, Emad Shash^*^

***Corresponding author:**

- **Affiliation:** National Cancer Institute - Breast Cancer Comprehensive Center, Medical Oncology Department, Cairo University, Cairo, Egypt
- **E-mail address:** [emad.shash@nci.cu.edu.eg](mailto:emad.shash@nci.cu.edu.eg)

**Supplementary Material 2. Patient Support Entities on Social Media (Facebook) - Pages**

| Name | Starting date | No. of followers | Type of page | Objective | Type of posts | Active/Not active | Qualification |
| --- | --- | --- | --- | --- | --- | --- | --- |
| King Hussein Cancer Foundation and Center مؤسسة ومركز الحسين للسرطان  <https://www.facebook.com/KingHusseinCancerFoundationandCenter> | September 6, 2009 | 770K | NGO |  | Specific | Active | Qualified |
| علاجات للسرطان Treatments for cancer  <https://www.facebook.com/profile.php?id=100064876860371> | October 13, 2010 | 10K | Community |    | Specific | Not active | Not qualified |
| الجمعية المصرية لدعم مرضى السرطان  The Egyptian Association for Cancer Patients Support  <https://www.facebook.com/CanSurvive> | May 16, 2011 | 299K | NGO. |  | General | Active | Qualified |
| Friends of Cancer Patients  <https://www.facebook.com/FOCPUAE> | May 22, 2011 | 6.4K | NGO. |    | Specific | Active | Qualified |
| المؤسسة المصرية لمكافحة سرطان الثدي  The Egyptian Foundation for Breast Cancer Prevention  <https://www.facebook.com/BCFEgypt> | May 20, 2012 | 87K | NGO. |  | General | Active | Qualified |
| المعهد القومي للأورام  National Cancer Institute  <https://www.facebook.com/NCI.eg?mibextid=V3Yony> | July 5, 2012 | 832K | Governmental. |  | General | Active | Qualified |
| Anti-Cancer Team (ACT)  <https://www.facebook.com/ACT.AUC> | September 6, 2012 | 4.7K | Community |  | General | Not Active | Not qualified |
| مرضى السرطان  Cancer Patients  <https://www.facebook.com/asaratan> | April 13, 2013 | 10K | Community |  | General | Not Active | Not Qualified |
| مرضى السرطان  Cancer Patients  <https://www.facebook.com/MARDA.ASARATAN> | May 2, 2013 | 12K | Community |  | Specific | Not active | Not qualified |
| جمعية أصدقاء المبادرة القومية ضد السرطان  Association of Friends of the National Cancer Institute  <https://www.facebook.com/AFNCI.officialpage> | May 23, 2013 | 1.9M | NGO. |  | General | Active | Qualified |
| السرطان ماكنش اختياري  Cancer was not my choice  <https://www.facebook.com/57cancer> | July 8, 2013 | 1.1K | Community | 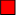   | Specific | Not active | Not qualified |
| جمعية الأمل لمكافحة السرطان  The Hope Association for Cancer Control  <https://www.facebook.com/alamlsy> | October 2, 2013 | 23K | Charity organization. |  | Specific | Active | Qualified |
| Saudi Cancer Foundation  <https://www.facebook.com/SaudiCancerF> | February 24, 2014 | 3.9K | NGO. |  | Specific | Not active | Qualified |
| Egyptian Cancer Survivors مصريون ضد السرطان  <https://www.facebook.com/EgyptianCancerSurvivors> | June 15, 2014 | 2.7K | Community |  | General | Not Active | Semi-Qualified |
| مكافحة مرض السرطان  Fighting Cancer  <https://www.facebook.com/profile.php?id=100067938694656> | July 23, 2014 | 1.1K | Community |    | Specific | Not active | Not qualified |
| نريد حياة  We want life  <https://www.facebook.com/wewantlife2017> | October 12, 2014 | 35K | NGO |  | General | Active | Qualified |
| علاج طبيعي للسرطان بالأعشاب الطبية  Natural Herbal Treatment for Cancer  <https://www.facebook.com/profile.php?id=100066509228196> | November 11, 2014 | 4.1K | Community |  | Specific | Not active | Not qualified |
| Shefaa Al Orman Hospital مستشفى شفاء الأورمان  <https://www.facebook.com/shefaalorman> | February 4, 2015 | 218K | NGO. |    | Specific | Active | Qualified |
| مؤسسة بهية  Baheya Foundation  <https://www.facebook.com/BaheyaFoundation> | February 17, 2015 | 4M | NGO. |  | General | Active | Qualified |
| مستشفى 500 500 لعلاج الأورام  500 500 Hospital for cancer treatment  <https://www.facebook.com/500500egypt> | July 8, 2015 | 449K | Governmental |  | General | Active | Qualified |
| الجمعية المصرية لصحة المرأة  The Egyptian Women's Health Association  <https://www.facebook.com/womenshealthegypt> | July 29, 2015 | 14K | NGO |  | General | Active | Qualified |
| حقيقة السرطان  The truth about cancer  <https://www.facebook.com/the.cancer.truth> | October 24, 2015 | 2.9K | Community |  | Specific | Not active | Not qualified |
| محاربي سرطان الدم  Blood Cancer Warriors  <https://www.facebook.com/Leukemia.survive> | November 4, 2015 | 3.4K | Community |  | General | Not active | Not qualified |
| جمعية الامل لرعاية مرضى السرطان  The Hope Association for Cancer Patients Care  <https://www.facebook.com/alamal.sohag> | May 10, 2016 | 4.5K | Community |  | Specific | Not active | Not qualified |
| قصتي مع السرطان  My Story with Cancer  <https://www.facebook.com/mystorywithcancer> | November 18, 2016 | 68K | Community |  | General | Not Active | Not qualified |
| حقائق عن مرض السرطان  Facts about cancer  <https://www.facebook.com/CancerFacts.InArabic> | November 23, 2016 | 5.2K | Community | 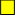 | Specific | Not active | Semi-Qualified |
| محاربين السرطان /Cancer  Warriors  <https://www.facebook.com/hassn.s999> | April 9, 2017 | 4.3K | Community | 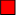   | specific | Not active | Not Qualified |
| وداعا للسرطان  Goodbye to Cancer  <https://www.facebook.com/profile.php?id=100060894100030> | May 11, 2017 | 1.1K | Community |  | Specific | Not active | Not qualified |
| محاربو السرطان Cancer Fighters  <https://www.facebook.com/CancerSupportC> | August 17, 2017 | 13K | Community |  | Specific | Active | Semi- Qualified |
| السرطان بدايه حياه_معا أقوي  Cancer is the beginning of a new life, together we are stronger  <https://www.facebook.com/CancerANewLifeBegins> | August 28, 2017 | 8.4K | Community | 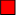   | specific | Not active | Semi- qualified |
| Cancer Killers  <https://www.facebook.com/profile.php?id=100080434007294> | September 14, 2017 | 1.6K | Community |  | Specific | Not active | Semi-qualified |
| مدد للدعم والإرشاد النفسي لمرضي السرطان  Madad: Support and Psychological Counseling for Cancer Patients  <https://www.facebook.com/Mdd.support> | September 15, 2017 | 6.8K | Community. |  | General | Not active | Semi-qualified |
| انا اقوي من السرطان  I am stronger than cancer  <https://www.facebook.com/Sooosooo123456> | November 21, 2017 | 3.9K | Community |    | Specific | Not active | Semi- qualified |
| وداعا للسرطان  Goodbye to cancer  <https://www.facebook.com/profile.php?id=100067326683819> | February 5, 2018 | 1.3K | Community |  | Specific | Not active | Not qualified |
| شعاع الأمل لمحاربي السرطان  Ray of Hope for Cancer Warriors  <https://www.facebook.com/brightofHope1> | July 2, 2018 | 3.1K | Community |  | Specific | Not active | Not qualified |
| مكافحة أمراض السرطان  Fighting Cancer Diseases  <https://www.facebook.com/Anti.cancer.H> | August 23, 2018 | 3.1K | Community |    | General | Not active | Not qualified |
| جمعية سحر الحياة لدعم ابطال السرطان  Sehr Al-Hayat Association for Supporting Cancer Champions  <https://www.facebook.com/www.sehralhyah> | September 18, 2018 | 10K | NGO. |  | Specific | Active | Qualified |
| تجربتي مع السرطان  My experience with cancer  <https://www.facebook.com/profile.php?id=100045651200231> | September 6, 2019 | 3.5K | Community |  | Specific | Not active | Not qualified |
| نحن اقوى من السرطان. صفحة لمساعدة مرضى السرطان طبيعيا  We are stronger than cancer. A page to help cancer patients naturally  <https://www.facebook.com/profile.php?id=100063748652639> | September 15, 2019 | 3.3K | Community | 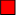 | Specific | Not active | Not Qualified |
| Cancer Care Egypt  <https://www.facebook.com/CancerCareEgypt> | April 19, 2020 | 100K | NGO |  | General | Active | Qualified |
| متعافية من السرطان  Recovered from cancer  <https://www.facebook.com/profile.php?id=100063232539005> | July 26, 2020 | 1.2K | Community |    | Specific | Not active | Not qualified |
| الجمعية العلمية للعلاج الموجه للسرطان  The Scientific Association for Targeted Cancer Therapy  <https://www.facebook.com/SATT.ORG2013> | December 14, 2021 | 7.9K | NGO. |  | Specific | Not active | Qualified |
| بيكم ومعاكم احنا أقوى من السرطان  With you and by your side, we are stronger than cancer  https://www.facebook.com/profile.php?id=100076638648265 | December 31, 2021 | 5.5K | Community. |  | General | Not Active | Semi-qualified |

**Note:** The figures presented in this table were revised and current as of September 29, 2023.

^*^NGO: Non-governmental organization.

General knowledge related to the disease.

Disease misconception correction.

Early detection awareness.

Psychological support.

Respond to patients inquires.

Free sessions/ workshops.

Help low-income patients financially (Donations).
